# Tumor cells express and maintain HMGB1 in the reduced isoform to enhance CXCR4-mediated migration

**DOI:** 10.3389/fimmu.2024.1358800

**Published:** 2024-05-13

**Authors:** Edisa Pirani, Philipp Paparoditis, Matteo Pecoraro, Gabriela Danelon, Marcus Thelen, Valentina Cecchinato, Mariagrazia Uguccioni

**Affiliations:** Laboratory of Chemokines in Immunity, Institute for Research in Biomedicine, Università della Svizzera italiana, Bellinzona, Switzerland

**Keywords:** CXCL12, HMGB1, heterocomplex, breast cancer, prostate cancer, thioredoxin

## Abstract

During inflammation and tissue regeneration, the alarmin High Mobility Group Box 1 (HMGB1), in its reduced isoform, enhances the activity of the chemokine CXCL12, forming a heterocomplex that acts via the chemokine receptor CXCR4. Despite the established roles of both HMGB1 and CXCL12 in tumor progression and metastatic spread to distal sites, the role of the CXCL12/HMGB1 heterocomplex in cancer has never been investigated. By employing a newly established mass spectrometry protocol that allows an unambiguous distinction between reduced (red-HMGB1) and oxidized (ox-HMGB1) HMGB1 isoforms in cell lysates, we demonstrate that human epithelial cells derived from breast (MCF-7 and MDA-MB-231) and prostate (PC-3) cancer predominantly express red-HMGB1, while primary CD3^+^ T lymphocytes from peripheral blood express both HMGB1 isoforms. All these cancer cells release HMGB1 in the extracellular microenvironment together with varying concentrations of thioredoxin and thioredoxin reductase. The CXCL12/HMGB1 heterocomplex enhances, via CXCR4, the directional migration and invasiveness of cancer cells characterized by high metastatic potential that possess a fully active thioredoxin system, contributing to maintain red-HMGB1. On the contrary, cancer cells with low metastatic potential, lack thioredoxin reductase, promptly uptake CXCL12 and fail to respond to the heterocomplex. Our study demonstrates that the responsiveness of cancer cells to the CXCL12/HMGB1 heterocomplex, resulting in enhanced cell migration and invasiveness, depends on the maintenance of HMGB1 in its reduced isoform, and suggests disruption of the heterocomplex as a potential therapeutic target to inhibit invasion and metastatic spread in cancer therapies.

## 1 Introduction

Over the years, extensive studies have shed light on the intricate interplay between tumor cells and the surrounding microenvironment. One pivotal aspect of this interaction involves chemokines, the molecular messengers orchestrating immune cell migration and maintaining immune system balance. Beyond their role in immunity, chemokines are present also in the tumor microenvironment and are produced both by cancer cells, tumor stroma and by tumor infiltrating leukocytes. The pattern of chemokines expressed in the tumor microenvironment can contribute to cancer progression and immune-mediate control. The chemokine CXCL12, and its corresponding receptor, CXCR4, are important players both in physiological and pathological conditions (1–4). In tumors, CXCL12 has been shown to promote vascularization and cell proliferation, while elevated expression of CXCR4 has been associated to the establishment of metastases in distal sites characterized by elevated levels of the chemokine (5–7). Among others, prostate and breast cancers preferentially metastasize to the bone and lungs via the CXCR4/CXCL12 axis, and approaches aimed at inhibiting this interaction have proven to significantly reduce metastatic spread (7–9).

High mobility group box 1 (HMGB1) is a protein that is retained in the nucleus by two lysine-rich nuclear localization sequences (NLS), where it is transiently associated with nucleosomes, and involved in DNA events that support genome stability, such as DNA repair, transcription, and telomere maintenance (10). It has been shown that during cellular death or inflammation, HMGB1 can also be passively or actively released into the extracellular milieu where it acts as damage-associated molecular pattern molecule (DAMP), binding to various receptors (10). Moreover, it contributes to tumor cell proliferation, invasion, and metastasis (11, 12), and increased expression of HMGB1 has been correlated with poor prognosis in various cancers (13, 14). In particular, the extracellular function of HMGB1 depends on the redox state of its three cysteines in position 23, 45 and 106 (15). Oxidized HMGB1, in which the cysteines in position 23 and 45 form a disulfide bond, activates Toll-like Receptor 2 (TLR2) and 4 (TLR4), and induces the release of pro-inflammatory chemokines and cytokines, which in turn trigger innate and adaptive immune cells (15). On the other hand, reduced HMGB1 binds both to the receptor for advanced glycation end products (RAGE), and in complex with CXCL12 to CXCR4, enhancing cell migration induced by this chemokine receptor (16). The redox status of HMGB1 is controlled by several mechanisms, including the thioredoxin system, which enables maintenance of HMGB1 in its reduced form (17). The oxidoreductase protein thioredoxin (Trx) and its reductase (TrxR), which regenerates active Trx, play a pivotal role in maintaining redox balance within cells, and in the extracellular space (17). In cancer, this system is frequently dysregulated, and correlates with tumor aggressiveness, therapy resistance, and poor prognosis (18–20).

While the individual functions of CXCL12 and HMGB1 in cancer have been extensively explored, the role of the CXCL12/HMGB1 heterocomplex remains unclear. Previous works have shown that the heterocomplex enhances tissue regeneration (21, 22), and leukocyte migration in sterile inflammation (16), and in active Rheumatoid Arthritis (17). In the present work, we established a protocol that, using mass spectrometry, allows an unambiguous distinction between the reduced (red-HMGB1) and oxidized (ox-HMGB1) HMGB1 redox isoforms. Human epithelial cells derived from breast (MCF-7 and MDA-MB-231) and prostate (PC-3) cancer express red-HMGB1 that is released in the extracellular microenvironment. When a fully active thioredoxin system is present, it contributes to maintain red-HMGB1. Directional migration and invasiveness of tumor cells is enhanced by the CXCL12/HMGB1 heterocomplex, and is CXCR4 dependent. Our study shows that, in addition to its function on immune cells, the CXCL12/HMGB1 heterocomplex fine tune modulates CXCL12 activity on tumor cells, and suggests disruption of the complex as a novel therapeutic strategy for inhibiting cancer cell migration and invasion.

## 2 Materials and methods

### 2.1 Reagents

Full-length HMGB1-Histidine-tagged protein was produced at the Institute for Research in Biomedicine Protein Facility under reducing conditions, and stored in phosphate-buffered saline (PBS). CXCL12 was chemically synthesized as previously described (23). CXCL12 Atto647-labeled (CXCL12-Atto647) and anti-CXCL12 Atto647-labeled antibody (clone K15C) were produced in-house (24).

### 2.2 Cell lines

The human epithelial breast cancer cell lines, MDA-MB-231 (CRM-HTB-26, ATCC) and MCF-7 (HTB-22, ATCC), were cultured in high glucose Dulbecco’s Modified Eagle Medium (DMEM, 61965-026, GIBCO, Thermo Fisher Scientific), supplemented with 10% fetal bovine serum (FBS) (16000-044, GIBCO, Thermo Fisher Scientific) and 1% penicillin-streptomycin (15070-063, GIBCO, Thermo Fisher Scientific). The human epithelial prostate cancer cell line, PC-3 (CRL-14355, ATCC), was cultured in RPMI 1640 Medium (31870-025, GIBCO, Thermo Fisher Scientific), supplemented with 10% FBS, 1% penicillin-streptomycin, and 1% GlutaMAX (35050-061, GIBCO, Thermo Fisher Scientific). Cells were detached using 2 mM ethylenediaminetetraacetic acid (EDTA)/PBS for 5 min under standard culture conditions.

### 2.3 qRT-PCR analysis

Cells were seeded in 24-well plates at 80% confluence. After 16 h, cells were washed with PBS in order to remove non-adherent cells and adherent cells detached for RNA extraction and qRT-PCR analysis. RNA was extracted using TRIzol Reagent (15596026, Invitrogen), and cDNA synthesized using Moloney Murine Leukemia Virus Reverse Transcriptase (M-MLV RT, 28025-013, Invitrogen) following the manufacturer’s instructions. q-PCR was performed using the 7500 Fast Real-Time PCR system (Applied Biosystems) with TaqMan™ Fast Universal PCR Master Mix (4352042, ThermoFisher Scientific) and the following set of probes: *CXCR4* (Hs00237052_m1), *CXCL12* (Hs00171022_m1), *HMGB1* (Hs1590761_g1), *TRX* (Hs01555214_g1), *TRXR1* (Hs00917067_m1), and *18S* (Hs99999901_s1).

### 2.4 Flow cytometric analysis

For quantification of CXCR4 surface expression, cells were stained for 20 min at 4°C using an anti-human CXCR4-PE conjugated antibody (clone 12G5, 306505, BioLegend), diluted 1:50 in FACS buffer (PBS with 1% FBS). To investigate the potential uptake of CXCL12 by the cells, CXCL12-Atto647 was added to the cell medium at the final concentration of 30 nM. After 45 min, the medium was discarded, cells were washed and resuspended in FACS buffer. To inhibit CXCR4 activity during CXCL12 uptake, cells were pre-treated with 10 μM AMD3100 (A5602, Sigma-Aldrich) for 1 h. CXCL12-Atto647 was then added to the cell medium at the final concentration of 30 nM and, after 45 min, cells were collected for flow cytometric analysis. Samples were acquired using the BD LSR Fortessa flow cytometer from BD Biosciences and analyzed with the FlowJo software (FLOWJO LLC). Relative Mean Fluorescence Intensity (rMFI) was calculated as the ratio between the MFI detected in the stained sample and the unstained control for each condition.

### 2.5 Confocal microscopy analysis

To assess CXCL12 and HMGB1 intracellular protein expression, cells were seeded in MatTek dishes. After 16 h, attached cells were washed with PBS, fixed with 4% paraformaldehyde in PBS for 10 min, permeabilized with 0.1% Triton X100 in PBS for 10 min, and stained for 2 h with a rabbit anti-HMGB1 antibody (clone EPR3507, ab79823, Abcam) and an anti-CXCL12 Atto647-labeled antibody (clone K15C). For HMGB1 detection, a goat anti-rabbit IgG-AlexaFluor488 conjugated secondary antibody (A-11070, Invitrogen), diluted 1:1000 in staining buffer (Dulbecco’s PBS, 0.01% sodium azide, 2% Bovine Serum Albumin, 5% goat serum), was used. Nuclei were counterstained with DAPI, and the cell membrane of cancer cells was stained using the MemBrite Fix cell surface staining kit from Biotium, following the manufacturer’s instructions. To examine the internalization of CXCL12, cells were seeded in MatTek dishes. After 16 h, attached cells were washed with PBS, and new medium containing 30 nM of CXCL12-Atto647 was added. After 45 min, cells were washed with PBS and fixed with 4% paraformaldehyde in PBS for 10 min. Nuclei were counterstained with 2 mM DAPI (62248, Thermo Fisher Scientific), and the cell membrane was stained using the MemBrite Fix cell surface staining kit from Biotium, following the manufacturer’s instructions. Finally, cells were analyzed using the Leica TCS SP5 fluorescence confocal microscope.

### 2.6 ELISA

The detection of HMGB1, thioredoxin, thioredoxin reductase 1, or CXCL12 in cancer cell supernatants was performed by ELISA following the manufacturer’s instructions: HMGB1 Express ELISA kit (30164033, TECAN), Trx (EK1254, Boster Biological Technology Co), TrxR1 (NBP2-68164, Novus Biologicals), and CXCL12 (DY350, R&D Systems). For these experiments, cells were seeded in 24-well plates at 80% confluence and after 16 h the cell supernatant was collected to determine HMGB1, Trx, TrxR1 or CXCL12 concentration.

### 2.7 CD3^+^ T-cell isolation

Blood samples from healthy donors were received as buffy-coats from the Central Laboratory of Swiss Red Cross (Basel, Switzerland), or the Centro Trasfusionale (Lugano, Switzerland), and usage approved by the Ethical Committee of the Canton Ticino (CE-3428). All samples were processed within 24 h after blood withdrawal. Peripheral blood mononuclear cells (PBMCs) were isolated using Ficoll-hypaque density centrifugation. CD3^+^ T-cells were isolated by negative immunoselection (130-096-535, human Pan T cell Isolation kit, Miltenyi Biotec) according to the manufacturer’s instructions. 8-10x10^6^ isolated CD3^+^ T-cells were washed twice with PBS, flash frozen, and stored at −80°C until processed for mass spectrometry analysis.

### 2.8 Liquid chromatography-tandem mass spectrometry

#### 2.8.1 Protein extraction and enzymatic digestion

Recombinant full length HMGB1-Histidine-tagged was used to set-up the LC-MS/MS protocol for the analysis of the redox status of the protein. HMGB1 (1 μg) was dissolved in 50 mM ammonium bicarbonate (ABC) buffer containing 8 M urea and sonication was performed in a Bioruptor (Diagenode, 15 cycles, 30s on, 30s off, high mode). Reduced cysteines were alkylated with 50 mM iodoacetamide (IAA-light) for 30 min at room temperature (RT). Proteins were precipitated over night with 80% Acetone at -20°C. Dried pellets were dissolved in 8 M urea in 50 mM ABC, sonicated and incubated with 10 mM dithiothreitol (DTT) for 20 min at RT in order to reduce previously oxidized cysteines. Newly reduced cysteines were alkylated with 50 mM isotopically labelled iodoacetamide-^13^C_2_, 2-D_2_ (IAA-heavy) for 30 min at RT. Digestion was carried out in 8 M urea in 50 mM ABC, overnight at RT with LysC (Wako Fujifilm, 1:100 w/w). Protease activity was stopped by adding 2% acetonitrile (ACN) and 0.3% trifluoroacetic acid (TFA). The samples were subsequently cleared by centrifugation for 5 min. The resulting peptides were purified by loading the supernatant into C18 StageTips (25), and eluted with 80% ACN, 0.5% acetic acid. Finally, the elution buffer was eliminated by vacuum centrifugation and purified peptides were resolved in 2% ACN, 0.5% acetic acid, 0.1% TFA for single-shot LC-MS/MS measurements. To determine the redox status of endogenous HMGB1 in the different cancer cell types, for each biological replicate, a cell pellet of approximately 4x10^6^ cells was washed twice in PBS, flash frozen and stored at -80°C. Cell lysis and protein extraction was performed in 8 M urea in 50 mM ABC by sonication. Alkylation, enzymatic digestion and peptide purification were performed as for the recombinant protein.

#### 2.8.2 LC-MS/MS analysis

Peptides were separated on an EASY-nLC 1200 HPLC system (Thermo Fisher Scientific) coupled online via a nanoelectrospray source (Thermo Fisher Scientific) to a Q Exactive HF mass spectrometer (Thermo Fisher Scientific). Peptides were loaded in buffer A (0.1% formic acid) into a 75 µm inner diameter, 50 cm length column in-house packed with ReproSil-Pur C18-AQ 1.9 µm resin (Dr. Maisch HPLC GmbH), and eluted over a 150-min linear gradient of 5 to 30% buffer B (80% ACN, 0.1% formic acid) at a 250 nl/min flow rate. The Q Exactive HF was operated in a data-dependent mode with the Xcalibur software (Thermo Scientific), with a survey scan range of 300-1,650 m/z, resolution of 60,000 at 200 m/z, maximum injection time of 20 ms and AGC target of 3e6. The top-10 most abundant ions with charge 2-5 were isolated with a 1.8 m/z isolation window and fragmented by higher-energy collisional dissociation (HCD) at a normalized collision energy of 27. MS/MS spectra were acquired with a resolution of 15,000 at 200 m/z, maximum injection time of 55 ms and AGC target of 1e5. Dynamic exclusion was set to 30s to reduce repeated sequencing.

#### 2.8.3 LC-MS/MS data analysis

MS raw files were processed using the MaxQuant software v.1.6.7.0 (26). The integrated Andromeda search engine (27) was employed to search spectra against the Human UniProt database (June 2019), and a common contaminants database (247 entries) to identify peptides and proteins with a false discovery rate of < 1%. Enzyme specificity was set as “LysC/P” with a maximum of 2 missed cleavages and minimum length of 6 amino acids. N‐terminal protein acetylation, methionine oxidation, and cysteine carbamidomethylation and heavy carbamidomethylation (^13^C_2_, 2-D_2_) were set as variable modifications. Match between runs was used to transfer identifications across samples based on mass and normalized retention times, with a matching time window of 0.7 min and an alignment time window of 20 min. Label‐free protein quantification (LFQ) was performed with the MaxLFQ algorithm (28) with a minimum required peptide ratio count of 1. Data obtained from the “modificationSpecificPeptides.txt” output table were analyzed by summing the intensities of all the peptides, with 0, 1 or 2 missed cleavages, containing either the light or heavy iodoacetamide derivative-modified form of each of the HMGB1cysteines, and referring it to the total peptides intensity for the specific position.

### 2.9 Scratch wound assay

PC-3 cells were grown to 100% confluency in a 96-well plate, and scratches were performed using a sterile 10 μL pipette tip. After the scratch, attached cells were washed with PBS, and fresh culture medium containing HMGB1-Histidine-tagged at 300 nM, CXCL12 at 10 nM or 100 nM, or the CXCL12/HMGB1 heterocomplex formed with CXCL12 at 10 nM and HMGB1-Histidine-tagged at 300 nM, was added. The ability of cells to migrate into the scratch area was monitored by recording the scratched area every 30 min for 4 h, using the ImageXpress Micro 4 Imager equipped with an incubation system set to CO_2_ 5%, O_2_ 95%, 37°C, and with a 4× objective. The area covered by migrated cells, either unstimulated or stimulated with HMGB1, CXCL12, or the CXCL12/HMGB1 heterocomplex, was calculated as μm^2^. To inhibit HMGB1-induced wound healing, the following reagents were added to the medium: AMD3100 (1 μM; A5602, Sigma-Aldrich), glychyrrizin (200 μM; 50531, Sigma-Aldrich) or a neutralizing antibody targeting CXCL12 (1 μg/ml; clone K15C). Cells were pre-treated with *Bordetella pertussis* toxin (1 μg/ml; P-7208, Sigma-Aldrich) for 30 min, washed once with PBS and fresh culture medium containing HMGB1 at 300 nM was added. MCF-7 and MDA-MB-231 cells were not suitable to perform wound healing assay, since they either tend to grow on multiple layers at confluence or easily detach from the well plate when the scratch is performed.

### 2.10 Chemotaxis assay

Directional migration of MDA-MB-231 cells was measured using the μ-Slide chemotaxis system from Ibidi (80326), following the manufacturer’s instructions and performed as previously described (29). Briefly, 6 μL of a suspension of MDA-MB-231 cells at a concentration of 4x10^6^ cells/mL in high glucose DMEM supplemented with 1% FBS (chemotaxis medium) was applied onto the “filling port” that represents the observation area. After cell adherence, the left reservoirs were filled with chemotaxis medium. The right reservoirs were filled with chemotaxis medium as control or with the same medium containing one of the following stimuli: HMGB1-Histidine-tagged at 300 nM, CXCL12 at 3 nM, 10 nM or 100 nM, or the CXCL12/HMGB1 heterocomplex formed with CXCL12 at 3 nM, 10 nM or 100 nM and HMGB1-Histidine-tagged at 300 nM. Phase contrast images were recorded every 10 min for 18 h using the ImageXpress Micro 4 Imager equipped with an incubation system set to CO_2_ 5%, O_2_ 95%, 37°C, and with a 4× objective. Single-cell tracking was performed by selecting the center of mass in each frame using the manual tracking plug-in tool for the ImageJ software. Spider plots representing the trajectories of tracked cells, forward migration indexes, and accumulated distances were obtained using the chemotaxis and migration plug-in tool (https://ibidi.com/chemotaxis-analysis/171-chemotaxis-and-migration-tool.html) from Ibidi.

### 2.11 Invasion assay

MDA-MB-231 and MCF-7 cell invasion was assessed using 24-well plate Corning Matrigel Invasion chambers with 8.0 μm pores (354480). 2x10^4^ cells were seeded in the upper well in 250 μL high glucose DMEM supplemented with 2% FBS. The bottom well was filled with 750 μL high glucose DMEM supplemented with 10% FBS, containing one of the following stimuli: HMGB1-Histidine-tagged at 300 nM, CXCL12 at 10 nM, or the CXCL12/HMGB1 heterocomplex formed with CXCL12 at 10 nM and HMGB1-Histidine-tagged at 300 nM. After 48 h, 4% paraformaldehyde in PBS was added for 30 min at 37°C, followed by a wash with DPBS. Wells were incubated for 15 min at room temperature with a blocking solution (DPBS, 2% BSA, 5% goat serum). The Matrigel containing cells was stained with a primary rabbit anti-collagen IV antibody (ab19808; Abcam, 1:1000 dilution), followed by a secondary goat anti-rabbit IgG AlexaFluor633-conjugated antibody (A21070, Invitrogen, 1:2000 dilution) in DPBS, 2% BSA, 0.005% Tween20 for 1 h at room temperature. Cell cytoskeleton was stained with Phalloidin-Atto565 (94072, Sigma Aldrich, 1:50), and nuclei counterstained with 2 mM DAPI (62248, Thermo Fisher Scientific). Finally, membranes were cut, mounted with SlowFade™ Diamond Antifade Mountant (S36967, Invitrogen), and z-stacks covering 120 μm were acquired using a Leica Stellaris 8 confocal microscope. Invaded cells were quantified using Imaris Microscopy Image Analysis Software, based on their position in the Matrigel.

### 2.12 Statistical analysis

**S**tatistical analyses were performed using GraphPad Prism Software (v.10.1). The statistical significance between two groups was assessed using unpaired, two tailed *t*-test. Differences among the cell lines were assessed using one-way ANOVA, followed by Tukey’s multiple comparison test. CXCL12-Atto674 uptake by cancer cells treated with the CXCR4 antagonist AMD3100 was analyzed using two-way ANOVA, followed by Šídák’s multiple comparisons test. Wound healing data were analyzed using two-way ANOVA, followed by Dunnett’s multiple comparison test. A *p* value below 0.05 was considered as significant.

## 3 Results

### 3.1 Presence and release of HMGB1 in breast and prostate cancer cell lines

With the aim of understanding the relevance of the CXCL12/HMGB1 heterocomplex in promoting tumor cell migration and invasion, we selected two breast (MDA-MB-231 and MCF-7) and one prostate (PC-3) cancer cell lines, characterized by different metastatic properties, for which the relevance of the CXCR4/CXCL12 axis in metastatic spread was previously established (30, 31). MDA-MB-231 and PC-3 cells are highly invasive and have a propensity to metastasize to distant organs. In contrast, MCF-7 cells derive from a less aggressive breast cancer with lower metastatic potential.

**Figure 1:**
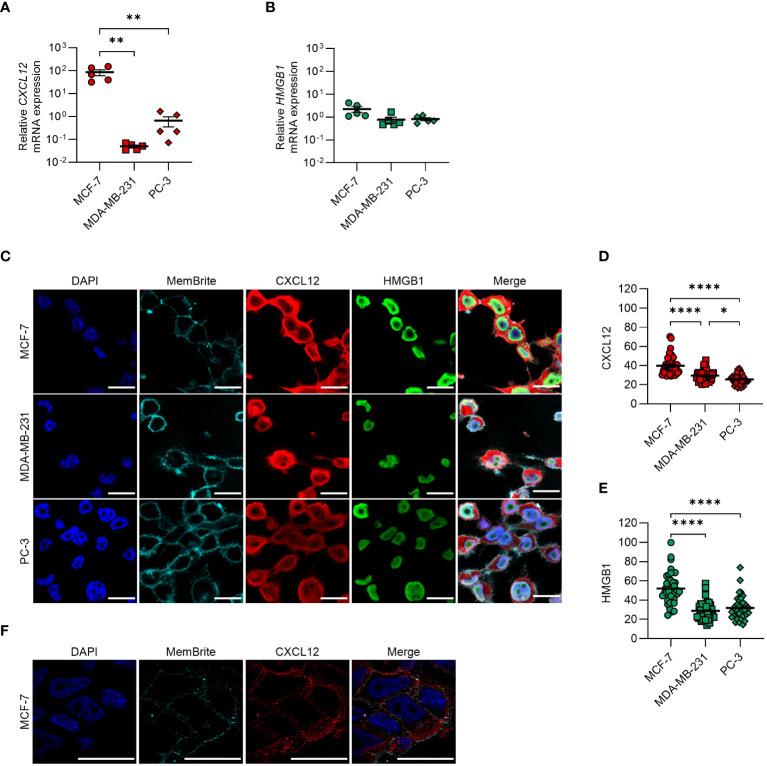
CXCL12 and HMGB1 are expressed in MCF-7, MDA-MB-231, and PC-3 cancer cell lines. **(A, B)** qRT-PCR analysis (normalized on *18S*) is shown for *CXCL12* and *HMGB1* mRNA in cancer cells. Data are shown as mean ± SEM of 5 independent experiments. Statistical analysis of the differences among the cell lines was performed using one way ANOVA, followed by Tukey’s multiple comparison test: **p < 0.01. **(C)** CXCL12 and HMGB1 expression assessed by confocal microscopy. In the merged images, CXCL12 is shown in red and HMGB1 in green. Nuclei and cell membranes were counterstained with DAPI (blue) and MemBrite Fix Dye (cyan), respectively. Representative images, of at least 3 independent experiments, are shown. Scale bar represents 20 µm. **(D, E)** CXCL12 **(D)** and HMGB1 **(E)** expression was evaluated in at least 40 cells and expressed as fluorescence intensity. Horizontal lines represent means. Statistical analysis of the differences among the cell lines was performed using one way ANOVA, followed by Tukey’s multiple comparison test: *p < 0.05, ****p < 0.0001. **(F)** Confocal microscope z-stack overlays showing the intracellular localization of CXCL12 in MCF-7 cells (scale bar 20 μm). CXCL12 is shown in red, while nuclei were counterstained with DAPI (blue) and cell membranes with the MemBrite Fix Dye (cyan).

We detected mRNA expression of CXCL12 and HMGB1 (**Figures 1A, B**) and demonstrated by confocal microscopy the presence of both proteins in all cell lines, with MCF-7 expressing the highest levels (**Figures 1C–E**). Z-stack confocal images suggested CXCL12 localization within cytoplasmic vesicles (**Figure 1F**).

**Figure 2:**
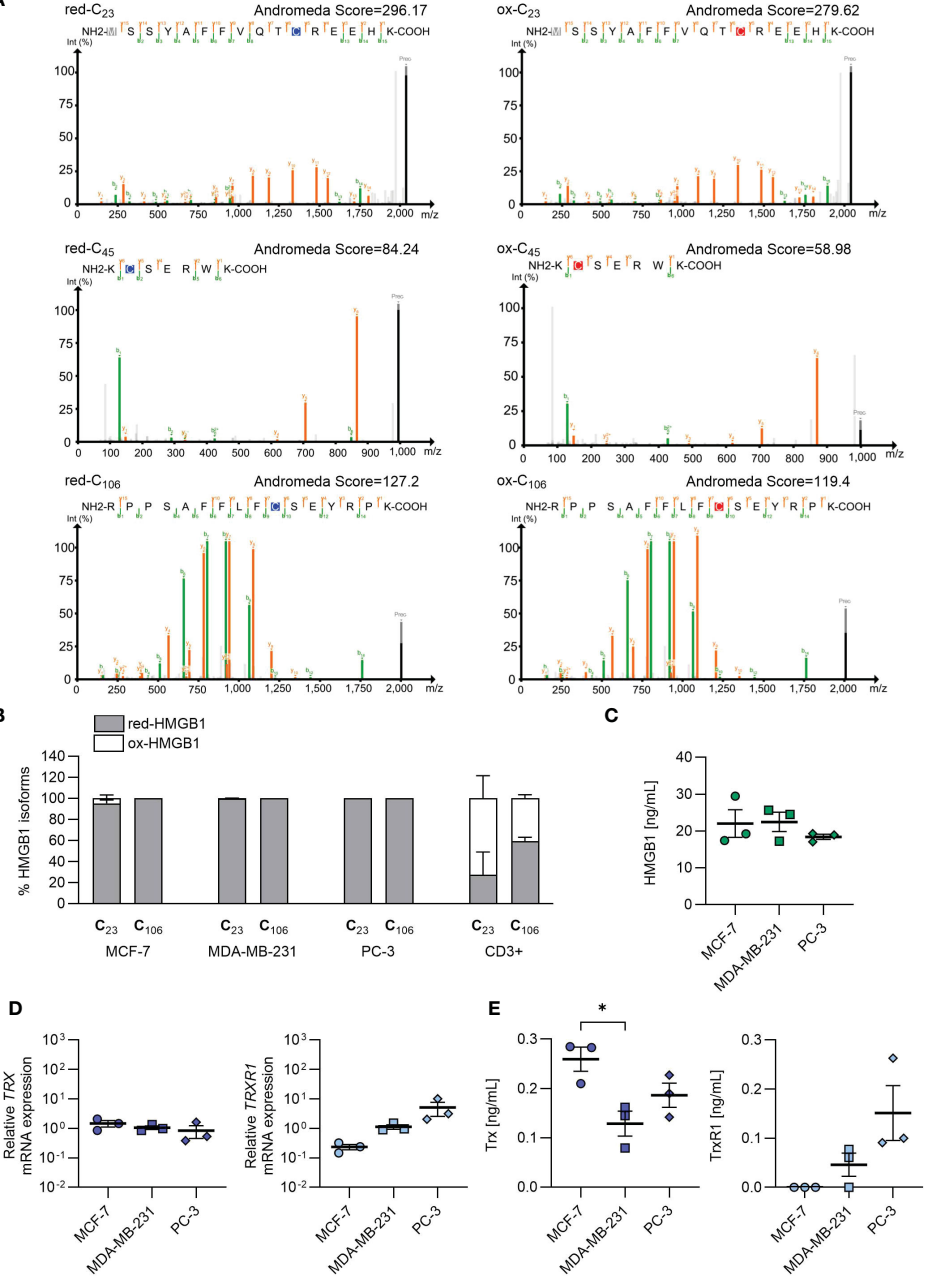
Endogenous redox status of HMGB1 in cancer cells, and release of HMGB1, Trx and TrxR1 in the extracellular milieu. **(A)** Annotated MS/MS spectra of the most abundant peptide forms containing either the light (blue) or heavy (red) iodoacetamide derivative-modified form of each of the HMGB1 cysteines identified with the recombinant full length HMGB1. Green and orange lines represent the annotated fragment peaks for each MS/MS spectrum, where green marks the b-ion series and orange marks the y-ion series. The resulting fragmentation pattern is reported on the peptide sequence with the same color code to visualize the peptide spectrum match. In grey the common methionine oxidation modification is depicted. Andromeda identifications are visualized with PDV (http://pdv.zhang-lab.org). **(B)** Percentage of reduced and oxidized HMGB1 based on the redox status of C23 and C106 in MCF-7 (n=3), MDA-MB-231 (n=3), PC-3 (n=3), and in CD3^+^ T cells from healthy donors (n=2). Data are shown as mean + SEM of the independent experiments performed. **(C)** HMGB1 concentration detected by ELISA in the cell culture supernatants. Data present the mean ± SEM of 3 independent experiments. **(D)** qRT-PCR analysis (normalized on *18S*) is shown for *TRX* and *TRXR1* mRNA in cancer cells. Data are shown as mean ± SEM of 3 independent experiments. **(E)** Concentration of Trx and TrxR1 detected by ELISA in the cell culture supernatants. Data present the mean ± SEM of 3 independent experiments. Statistical analysis of the differences among the cell lines was performed using one way ANOVA, followed by Tukey’s multiple comparison test: *p < 0.05.

Given the importance of the redox status of HMGB1 in enhancing chemokine-induced migration, we established a mass spectrometry protocol for determining the redox status of cell-endogenous HMGB1 (**Supplementary Figure 1**). When employing recombinant HMGB1, we were able to identify peptides containing each of the three cysteines (C23, C45 and C106) in both the reduced (red-HMGB1) or oxidized (ox-HMGB1) isoforms (**Figure 2A**). However, peptides containing cysteine 45 were always detected with intensity one or two orders of magnitude lower than the peptides encompassing cysteines 23 and 106, and generally with lower Andromeda identification scores. Moreover, the predominant peptides covering cysteine 45 contained an extra amino acid resulting from a missed cleavage at the N-terminus of the derivatized cysteine, hinting at a possible cleavage issue at that specific site. Consequently, when the analysis was performed on more complex cell lysates, the peptides containing the second cysteine (C45) were often undetected or detected only with very low intensity. Therefore, it was not possible to obtain conclusive information regarding the redox status of cysteine 45. Analyses with the alternative proteases ArgC and GluC (data not shown) also gave similar results, indicating that the detection of this region is particularly challenging, possibly due to its amino acid composition and physiochemical properties. Nevertheless, the presence of the peptides corresponding to the first and third cysteine (C23 and C106) allowed distinguishing the two isoforms. In all samples, we detected a predominant presence of intracellular red-HMGB1, while both HMGB1 isoforms were present in primary CD3^+^ T cells isolated from the bloodstream of healthy individuals (**Figure 2B**).

To investigate the capability of the cancer cells to release HMGB1 in the microenvironment, and maintain it in the reduced form, we measured HMGB1, Trx and TrxR1 concentration in cell supernatants after overnight culture. As shown in **Figure 2C**, MCF-7, MDA-MB-231 and PC-3 cells released similar amount of HMGB1. Different levels of Trx and TrxR1 were detected both in term of mRNA expression and of concentration in the cell supernatant derived from the three cell lines, indicating a distinct capability of the tumors analyzed to maintain HMGB1 in the reduced isoform (**Figures 2D, E**). In particular, the supernatants obtained from PC-3 and MDA-MB-231 cell cultures contained both Trx and TrxR1, while the supernatants from MCF-7 did not contained TrxR1 indicating an impairment of this cell line to regenerate Trx, and therefore to maintain HMGB1 in its reduced form.

The release of HMGB1 into the extracellular milieu, accompanied by the presence of distinctive levels of the thioredoxin system components, highlights the potential of some tumors, especially those with high metastatic properties to maintain HMGB1 in its reduced form. These observations set the stage for better understanding the role of the CXCL12/HMGB1 heterocomplex in promoting tumor migration and invasion.

### 3.2 A functional CXCR4 mediates CXCL12 uptake in cancer cells

**Figure 3:**
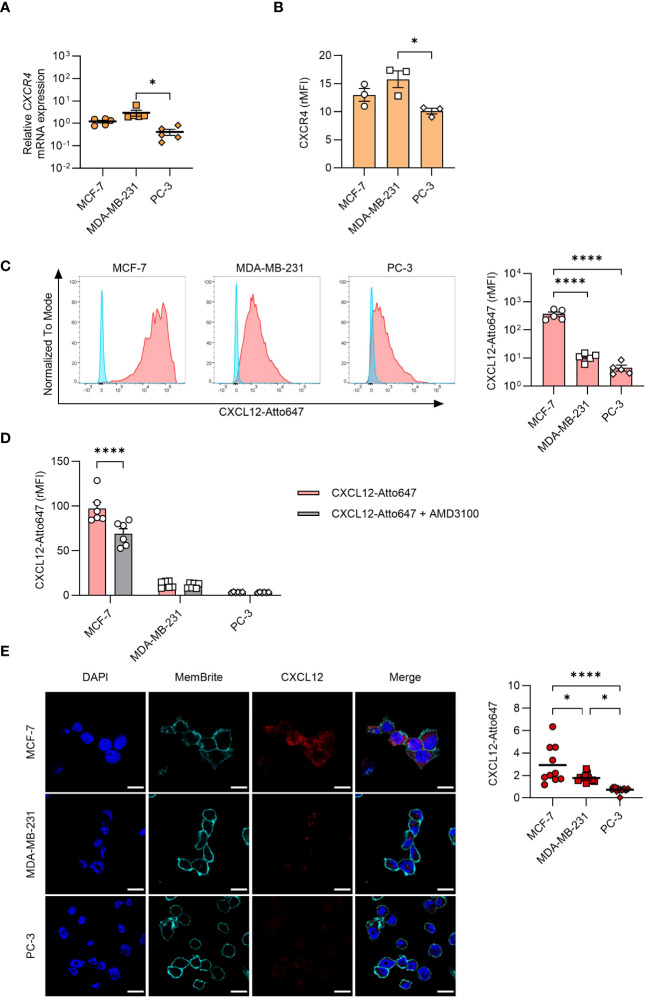
CXCL12 uptake by cancer cells. **(A)** qRT-PCR analysis (normalized on *18S*) is shown for *CXCR4* mRNA expression in cancer cells. Data are shown as mean ± SEM of 5 independent experiments. **(B)** CXCR4 expression analyzed by flow cytometry in MCF-7, MDA-MB-231, and PC-3 cells. Data are shown as relative MFI (rMFI, mean ± SEM) of 3 independent experiments. **(C)** Uptake of CXCL12-Atto647 by cancer cells analyzed by flow cytometry. Left: A representative histogram showing fluorescence intensity of unstimulated (blue) and CXCL12-Atto647 stimulated cells (red). Right: CXCL12-Atto647 uptake shown as rMFI (mean ± SEM) of 5 independent experiments. **(D)** Uptake of CXCL12-Atto674 by cancer cells treated with the CXCR4 antagonist AMD3100. Chemokine uptake shown as rMFI (mean ± SEM) of 6 independent experiments. Statistical analysis of the differences among the cell lines was performed using two-way ANOVA, followed by Šídák’s multiple comparisons test: ****p < 0.0001. **(E)** CXCL12-Atto647 uptake assessed by confocal microscopy in MCF-7, MDA-MB-231, and PC-3 cells. Left: In the merged images, CXCL12 is shown in red. Nuclei and cell membranes were counterstained with DAPI (blue) and MemBrite Fix Dye (cyan), respectively (scale bars: 20 μm). Right: CXCL12-Atto647 uptake was evaluated in at least 10 cells and expressed as fluorescence intensity. Horizontal lines represent the mean values. Statistical analysis of the differences among the cell lines was performed using one way ANOVA, followed by Tukey’s multiple comparison test: *p < 0.05, ****p < 0.0001.

Despite the abundant intracellular presence of CXCL12 detected by confocal microscopy, we could not detect the protein in cell supernatants (data not shown), raising the possibility for a rapid secretion-internalization loop. To explore the involvement of this mechanism, we assessed both CXCR4 expression (**Figures 3A, B**) and CXCL12 uptake by the different cell lines (**Figures 3C–E**). CXCR4 was expressed on all cell types, and they efficiently internalized CXCL12, with MCF-7 exhibiting a significantly higher uptake compared to the other cell types (**Figures 3A–D**). To corroborate these data, inhibition of CXCR4 with AMD3100 significantly reduced CXCL12 uptake in MCF-7 (**Figure 3D**). To exclude that the results obtained by flow cytometry were partially due to binding of the chemokine to the receptor or to the glycosaminoglycans (GAGs) on cell surface, acidic wash or a cocktail of enzymes shaving GAGs were applied before cytometry analysis (data not shown), supporting the results obtained. Moreover, the confocal microscopy analysis confirmed an active CXCL12 internalization process (**Figure 3E**).

### 3.3 CXCL12/HMGB1 activity on cancer cells

To gain insights into the relevance of the CXCL12/HMGB1 heterocomplex in cancer cell activities, we evaluated their capability to promote wound healing, migrate and invade the extracellular matrix.

**Figure 4:**
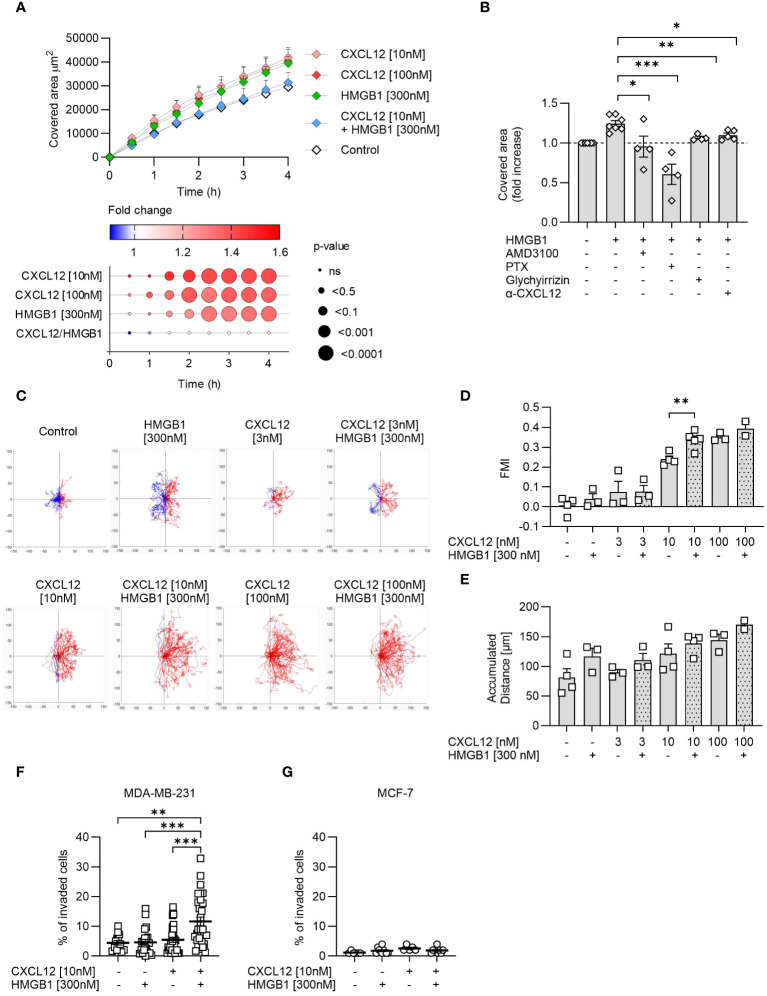
The CXCL12/HMGB1 heterocomplex enhances cancer cells wound healing, migration and invasion. **(A)** Wound healing in response to CXCL12, HMGB1 or the CXCL12/HMGB1 heterocomplex in PC-3 cells. Top panel: data are shown as area covered by migrating cells over time and represent the mean + SEM of at least 3 independent experiments. Bottom panel: dot plot representing statistical differences in covered area over time by stimulated cells compared to control cells. Circle size represents statistics; colors represent the fold-change increase (red) or decrease (blue), shown as ratio of the covered area of stimulated over control cells. Statistical analysis was performed using two-way ANOVA, followed by Dunnett’s multiple comparison test. **(B)** Inhibition of HMGB1-induced wound healing by AMD3100, *Bordetella pertussis* toxin (PTX), glychyrrizin and a neutralizing antibody targeting CXCL12 (α-CXCL12). Data are shown as fold increase of the covered area after 3 h compared to unstimulated PC-3 cells. Statistical analysis was performed using unpaired t-test: *p < 0.05, **p < 0.01, ***p < 0.001. **(C)** Cell migration assay. MDA-MB-231 cells were allowed to migrate in response to the indicated concentrations of CXCL12 and/or HMGB1 for 18 h. Representative spider plots, showing the trajectories of at least 60 single tracked cells migrating following (red) or moving in the opposite direction (blue) of the gradient, are shown. Blue and red dots in the plots represent the final position of each single tracked cell. **(D)** Quantitative evaluation of the Forward Migration Index (FMI) is shown. Data represent the mean + SEM of at least 2 independent experiments. **(E)** Quantitative evaluation of the accumulated distance from the experiments shown in **(D)**. Statistical analysis was performed using unpaired t-test: **p < 0.01. **(F, G)** Invasion assay. Percentage of MDA-MB-231 **(F)** or MCF-7 **(G)** cells invading the matrigel 48 h after stimulation with the indicated concentrations of CXCL12 and/or HMGB1 in at least 5 fields. Horizontal lines represent means ± SEM. Statistical analysis was performed using one-way ANOVA, followed by Tukey’s multiple comparison test: **p < 0.01, ***p < 0.001.

Among the three cancer cell lines, PC-3 cells because of their growth characteristics are the most suitable to assess wound healing in response to chemotactic stimuli. CXCL12 significantly enhanced wound healing compared to control at both concentrations used (10 nM and 100 nM), with the effect starting 1 hour after triggering (**Figure 4A**). A similar enhancement was achieved with the addition of HMGB1 alone but at a later time point, while surprisingly the CXCL12/HMGB1 heterocomplex did not have any effect on wound healing (**Figure 4A**). These findings prompted us to hypothesize that release of CXCL12 from PC-3 could account for the response observed with HMGB1 alone, through the formation of the heterocomplex. Indeed, AMD3100, a CXCR4 antagonist, and the *Bordetella pertussis* toxin (PTX), inhibiting Gαi of the G-protein coupled receptors, abolished this enhancement, thus indicating that HMGB1 activity requires the CXCR4 receptor and a (GPCR)-dependent mechanism (**Figure 4B**). The involvement of CXCL12 in the activity observed using HMGB1 alone was further proven by using a neutralizing antibody against the chemokine, or glycyrrhizin, a known inhibitor of the heterocomplex (**Figure 4B**).

Cell migration was assessed in MDA-MB-231 cells, a line derived from a highly metastatic breast cancer, using a two-dimensional chemotaxis assay. The results showed that MDA-MB-231 migrated in response to CXCL12 in a dose-dependent manner and that HMGB1 was able to enhance the response to CXCL12 at the suboptimal concentration of 10 nM (**Figures 4C, D**), as observed in the past for primary leukocytes (16). Of note, the directional migration induced by the CXCL12/HMGB1 heterocomplex was comparable to the one observed in response to the highest CXCL12 concentration (100 nM, **Figures 4C, D**). In addition, HMGB1 alone induced an increase, even if not reaching statistical significance, in the accumulated distance, a parameter that represents the length of the entire cell path regardless of directionality, resembling what observed for the PC-3 cells in the wound healing assay (**Figure 4E**).

The invasion assay was performed on MDA-MB-231 and MCF-7, the two breast-cancer cell lines that differ in their metastatic properties, and for their potential to maintain HMGB1 in the reduced form. MDA-MB-231 and MCF-7 did not invade the matrigel in response to CXCL12 at the sub-optimal concentration of 10 nM, while when the chemokine was combined with HMGB1, we observed a significant invasion of MDA-MB-231 but not of MCF-7 (**Figures 4F, G**).

Together, these results underscore how different cancer cells respond to the CXCL12/HMGB1 heterocomplex enhancing directional cell migration and invasion, which depends on the balance between maintaining HMGB1 in its reduced form and promptly removing CXCL12 from the microenvironment.

## 4 Discussion

Despite the well-established roles of CXCL12 and HMGB1 in cancer metastasis, the role of the CXCL12/HMGB1 heterocomplex has never been investigated. To determine the presence of a red-HMGB1 that can complex with CXCL12 in the tumor microenvironment, a mass spectrometry protocol that allows an unambiguous differentiation between the HMGB1 redox isoforms was developed.

Intracellular HMGB1 isolated from selected cancer cell lines is present as red-HMGB1, while both isoforms are present in primary CD3^+^ T cells purified from the blood of healthy donors. These data are in line with a previous publication that, by western blot analysis, associated the presence of ox-HMGB1 with leukocyte infiltration into the tumor (32). High concentrations of reactive oxygen species have been shown to induce C106-mediated anti-parallel HMGB1 dimerization to protect DNA from damage, both *in vitro* and *in vivo* (33). Of note, in CD3^+^ T cells, but not in the tumor cell lines, the cysteine in position 106 was either in the reduced or oxidized isoform, suggesting the presence of both HMGB1 monomers and dimers in these cells. Our results on the cancer cell line analyzed, are in line with previous studies demonstrating that monomeric HMGB1 is expressed by tumor cells, and dimerization is only induced upon exposure to therapeutic irradiation (33).

We found that HMGB1 is actively released by cancer cells together with the components of the thioredoxin system. Dysregulation of Trx and TrxR has been observed in numerous cancer types, including breast (34), lung (18), and colorectal cancer (19), among others. Increased expression and activity of these proteins have been associated with enhanced tumor growth and metastasis, increased resistance to chemotherapy, and poor prognosis (18–20). Of note, our data show that cells with high metastatic potential, such as MDA-MB-231 and PC-3, express and release both Trx and TrxR1, thus sustaining the maintenance of red-HMGB1 in the extracellular milieu and supporting the formation of the CXCL12/HMGB1 heterocomplex. On the contrary, cells with low metastatic potential, such as MCF-7, release only Trx, suggesting that HMGB1 can be easily oxidized in the microenvironment in the absence of TrxR1, which reduces Trx to be catalytically re-activated. In line with the different ability of the three cell lines to sustain the formation and activity of the CXCL12/HMGB1 heterocomplex, we demonstrated that the heterocomplex promotes invasion of MDA-MB-231, but not of MCF-7 cells, and that PC-3 cells migrate in response to HMGB1 alone, thanks to their release of CXCL12. These data further support previous findings by Bhatia et al., demonstrating the correlation between the expression of Trx1 and MDA-MB-231 cells capacity to invade *in vitro* (35).

Breast and prostate tumors can spread at distal sites via CXCR4, following local gradients of CXCL12 (7, 9). In order to determine whether, in the tumor microenvironment, tumor cells could be also source of CXCL12, we examined the mRNA and protein expression of CXCL12 in the three cell lines analyzed. We found discrepancies between CXCL12 mRNA and protein expression, which might be the results of multiple simultaneous processes, including chemokine secretion and uptake. PC-3 cells express intermediate levels of CXCL12 mRNA, the lowest levels of CXCL12 protein, uptake CXCL12 with low efficiency, possess a functional thioredoxin system, and respond to both CXCL12 and HMGB1 in the wound healing assay. The HMGB1-mediated PC-3 migration we observed is inhibited by *Bortedella pertussis* toxin, AMD3100, a neutralizing anti-CXCL12 antibody and glycyrrhizin, indicating that this effect occurs via CXCR4 through the production of CXCL12 and formation of the CXCL12/HMGB1 heterocomplex in the microenvironment. Exogenous addition of the heterocomplex does not promote wound healing, in the timeframe analyzed which can be explained by the fact that these cells release both CXCL12 and HMGB1, and therefore the concentration of the heterocomplex in the medium is above optimal. We have previously demonstrated that both monocytes and HeLa cells respond to increasing concentrations of the CXCL12/HMGB1 heterocomplex following a bell-shaped curve (16, 29). An excessive triggering of CXCR4, by high concentration of the heterocomplex or the chemokine alone, might induce receptor internalization and degradation and therefore impair cell migration (29).

Cell migration and invasiveness in the presence of the gradient, better recapitulate the metastatic process. MDA-MB-231 cells express the lowest CXCL12 mRNA and intermediate CXCL12 protein levels, have intermediate ability to uptake exogenous CXCL12, and do not migrate or invade in response to HMGB1 alone. These data imply that either the low concentration of the chemokine produced is retained inside the cells, or upon release promptly uptaken. As a result, the formation of the CXCL12/HMGB1 heterocomplex would be possible thanks to the release of CXCL12 by other cell types in the tumor microenvironment. Indeed, these cells are able to migrate and invade in the presence of exogenously provided CXCL12, and more importantly, these activities are enhanced in the presence of the heterocomplex, thanks to the release of both components of the thioredoxin system. MCF-7 cells uptake very efficiently the chemokine, release HMGB1, but do not have a fully functional thioredoxin system. The combination of these mechanisms could explain the inability to invade both in the presence of CXCL12 alone or the heterocomplex. CXCL12 uptake by MCF-7 cells was significantly but not completely blocked by inhibiting CXCR4 with AMD3100, suggesting that other receptors, such as the scavenger receptor ACKR3, might be involved in removing the chemokine from the micro-environment (36). The expression of functionally active ACKR3 has been previously described not only in MCF-7 cells (37), but also in MDA-MB-231 (38) and PC-3 cells (39).

Inhibition of TrxR1 has emerged as a potential therapeutic strategy in cancer treatment. This inhibition results in increased levels of ROS, cell apoptosis, necrosis, and autophagy (40–42). As an example, auranofin has been identified as a promising candidate to be combined to chemotherapy and radiation therapy, as it has been shown to inhibit cancer cell growth in melanoma (43) and breast cancer (44). Auranofin was included in a recent clinical trial (NCT01737502) targeting advanced or recurrent non-small cell lung or small cell lung cancers. TrxR inhibition might additionally promote oxidation of HMGB1, and the subsequent inhibition of the CXCL12/HMGB1 heterocomplex formation in the tumor microenvironment.

The present work shed light on the activity of the CXCL12/HMGB1 heterocomplex on cancer cells, particularly those with a high metastatic potential and a functional thioredoxin system. Enhancement of cell invasiveness and metastasis, supported by the heterocomplex can only occur in the presence of an extracellular microenvironment able to maintain HMGB1 in the reduced isoform. Given the role of the CXCR4/CXCL12 axis in breast cancer metastatic spread, and the differences observed between MDA-MB-231 and MCF-7 cells, our data point to initial stages of breast cancer as possible models where targeting the CXCL12/HMGB1 heterocomplex could be particularly promising. Further *in vivo* studies are needed to validate the present results and assess the disruption of the heterocomplex as a potential target in cancer therapies for inhibiting invasion and metastatic spread.

## Data availability statement

The mass spectrometry proteomics data have been deposited to the ProteomeXchange Consortium via the PRIDE partner repository with the dataset identifier PXD048091 (http://www.ebi.ac.uk/pride/archive/projects/PXD048091).

## Ethics statement

The studies involving humans were approved by Ethical Committee Canton Ticino (Switzerland). The studies were conducted in accordance with the local legislation and institutional requirements. The participants provided their written informed consent to participate in this study.

## Author contributions

EP: Data curation, Formal analysis, Investigation, Visualization, Writing – original draft. PP: Data curation, Formal analysis, Investigation, Visualization, Writing – review & editing. MP: Formal analysis, Investigation, Methodology, Visualization, Writing – review & editing. GD: Investigation, Writing – review & editing. MT: Resources, Writing – review & editing. VC: Conceptualization, Data curation, Formal analysis, Visualization, Writing – original draft, Supervision. MU: Conceptualization, Funding acquisition, Project administration, Supervision, Writing – original draft.
