# Supplementary material for: Tumor cells express and maintain HMGB1 in the reduced isoform to enhance CXCR4-mediated migration
Source: Front Immunol. 2024 May 13;15:1358800. doi: 10.3389/fimmu.2024.1358800 (PMC11128625; doi:10.3389/fimmu.2024.1358800)
Supplement: Supplementary file 1 [file DataSheet_1.pdf]

Supplementary Material

Tumor cells express and maintain HMGB1 in the reduced isoform to enhance CXCR4-mediated migration

Edisa Pirani[1], Philipp Paparoditis[1#], Matteo Pecoraro[1], Gabriela Danelon[1], Marcus Thelen[1], Valentina Cecchinato[1§*], Mariagrazia Uguccioni[1§*]

[1]Laboratory of Chemokines in Immunity, Institute for Research in Biomedicine, Università della Svizzera italiana, Bellinzona, Switzerland.

[#]Present address: Department of Immunology, Weizmann Institute of Science, Rehovot, Israel.

[§]These authors contributed equally to this work and share senior authorship.

*** Correspondence:**

Valentina Cecchinato, Institute for Research in Biomedicine, Università della Svizzera italiana, Via Chiesa 5, 6500 Bellinzona, Switzerland
valentina.cecchinato@irb.usi.ch

Mariagrazia Uguccioni, Institute for Research in Biomedicine, Università della Svizzera italiana, Via Chiesa 5, 6500 Bellinzona, Switzerland
mariagrazia.uguccioni@irb.usi.ch

A

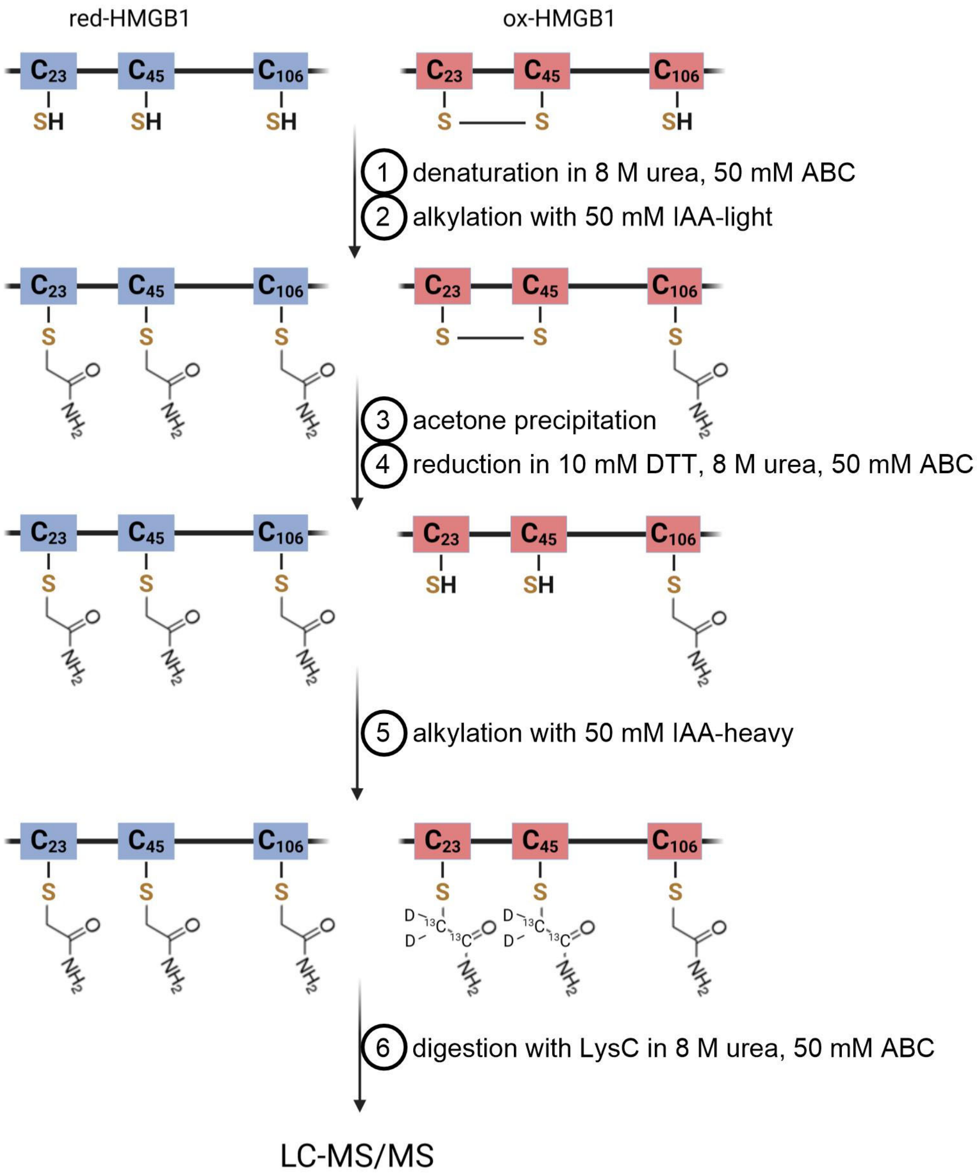

Supplementary Figure 1. Schematic representation of Liquid Chromatography-tandem Mass Spectrometry protocol to determine HMGB1 redox isoforms. ABC: ammonium bicarbonate buffer; DTT: dithiothreitol; IAA-light: iodoacetamide; IAA-heavy: isotopically labelled iodoacetamide-$^{13}C_2$, 2-D_2.
